# Intrinsic Activity Reductions and Cerebello‐Cortical Diaschisis‐Like Mechanism in Friedreich Ataxia: An fALFF Resting‐State fMRI Study

**DOI:** 10.1002/brb3.71866

**Published:** 2026-10-28

**Authors:** Naeije Gilles, Trotta Nicola, Rovai Antonin

**Affiliations:** ^1^ Department of Neurology Université Libre de Bruxelles (ULB), Hôpital Universitaire de Bruxelles (H.U.B.), CUB Hôpital Erasme Brussels Belgium; ^2^ UNI–ULB Neuroscience Institute, Laboratoire de Neuroanatomie et Neuroimagerie translationnelles (LN^2^T) Université Libre de Bruxelles (ULB) Brussels Belgium; ^3^ Department of Translational Neuroimaging Université Libre de Bruxelles (ULB), Hôpital Universitaire De Bruxelles (H.U.B.), CUB Hôpital Erasme Brussels Belgium

**Keywords:** ataxia, cerebellar atrophy, cerebellum, cognition, cognition, cognitive psychology, Friedreich Ataxia, gray matter, neuroscience, resting‐state fMRI

## Abstract

**Background:**

Friedreich ataxia (FA) is characterized by progressive degeneration of the cerebellum and its output pathways, particularly the dentato–thalamo–cortical system, leading to motor impairment and the cerebellar cognitive affective syndrome (CCAS). Resting‐state fMRI studies in FA have focused on alterations of long‐range functional connectivity, but little is known about how intrinsic regional neural activity is affected within cerebello‐cortical networks.

**Methods:**

Twenty‐eight adults with genetically confirmed FA and 28 age‐ and sex‐matched healthy controls underwent resting‐state functional MRI. Intrinsic brain activity was quantified using the fractional amplitude of low‐frequency fluctuations (fALFF). Whole‐brain comparisons were performed between controls and patients, and between patients with definite CCAS (FA‐Cog) and cognitively preserved patients (FA‐NoCog). Within the FA group, voxelwise regressions assessed associations between fALFF, motor severity (Scale for the Assessment and Rating of Ataxia [SARA]), and cognitive performance (Cerebellar Cognitive Affective Syndrome Scale [CCAS‐S]), adjusting for age, sex, head motion, and cerebellar gray matter volume.

**Results:**

Compared with controls, patients exhibited reduced fALFF in medial and lateral prefrontal regions. Higher SARA scores were associated with lower fALFF in occipital, sensorimotor, posterior cingulate, and cerebellar regions, whereas higher CCAS‐S scores correlated with higher fALFF in bilateral prefrontal and cerebellar cognitive networks. Compared with FA‐NoCog, FA‐Cog patients showed lower fALFF in bilateral frontal and insular regions.

**Conclusions:**

Resting‐state fALFF reveals downstream dysfunction of distributed cerebello‐cortical networks in FA. Associations with motor severity and cognition, together with additional fronto‐insular dysfunction in patients with definite CCAS, support progressive cerebello‐cortical disconnection consistent with a diaschisis‐like mechanism.

## Graphical abstract

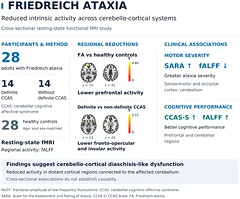

## 1 Introduction

Friedreich ataxia (FA) is the most common autosomal‐recessive ataxia and leads to progressive neurodegeneration within the cerebellum and its output pathways (Pandolfo 2008). Neuropathological studies consistently report severe involvement of the dentate nuclei and grumose degeneration of Purkinje cell terminals, which together produce a profound disruption of cerebellar efference through the dentato–thalamo–cortical (DTC) tract (Koeppen and Mazurkiewicz 2013). These projections target both primary motor cortices and widespread association areas (Guell et al. 2020), including dorsolateral and medial prefrontal regions. This could explain the gait and limb in FA but also a characteristic profile of executive, visuospatial, linguistic, and affective impairments grouped under the cerebellar cognitive affective syndrome (CCAS) (Destrebecq et al. 2023).

Although structural atrophy and tract degeneration are well documented in FA (Harding et al. 2021), far less is known about how spontaneous neural activity is altered along cerebello‐cortical systems in the disease. Resting‐state fMRI (rs‐fMRI) provides a noninvasive approach to study spontaneous brain activity in the absence of an explicit task. Most rs‐fMRI investigations have focused on functional connectivity, examining temporal correlations between BOLD signals in spatially distinct brain regions, characterizing networks that closely mirror task‐evoked systems such as the default mode, language, salience, and executive control networks (Smith et al. 2009); accordingly, much of the recent literature in neurodegenerative diseases and movement disorders has emphasized alterations in these interregional coupling patterns (Marapin et al. 2023; Hohenfeld et al. 2018).

However, rs‐fMRI also contains valuable information about local spontaneous activity. The fractional amplitude of low‐frequency fluctuations (fALFF) quantifies the power of slow BOLD oscillations relative to the total spectral power at each voxel, an approach specifically designed to emphasize meaningful low‐frequency fluctuations while reducing contributions from broadband noise, thereby providing a sensitive index of baseline signal amplitude in rs‐fMRI (Zuo et al. 2010; Zou et al. 2008; Zhang et al. 2021). Unlike functional connectivity, which captures temporal synchrony between spatially distributed regions, fALFF measures the local magnitude of spontaneous BOLD fluctuations (Deng et al. 2022), enabling the detection of regional hypoactivity even when connectivity appears preserved (Zang et al. 2007). Low‐frequency oscillations vary systematically across brain regions (Han et al. 2011), differ between individuals, and are altered in multiple neurological and psychiatric disorders: in Alzheimer's disease, for instance, fALFF values are lower in the posterior cerebellar and cortical associative areas involved in cognition and memory (Zhang et al. 2024). Simultaneous PET–fMRI studies have demonstrated strong associations between fALFF and regional glucose metabolism or cerebral blood flow, particularly within executive control and default mode networks (Aiello et al. 2015; Marchitelli et al. 2018; Riedl et al. 2014). Together, these observations support the view that fALFF indexes baseline neuronal and metabolic activity at the regional level conversely to long‐range functional connectivity. In FA, long‐range functional connectivity rs‐fMRI investigations disclosed reduced cerebello‐cortical functional connectivity in both motor and associative circuits (Kerestes et al. 2023), as well as disrupted Crus I–prefrontal connectivity in association to FA cognitive impairment (Destrebecq et al. 2025), consistently pointing to altered long‐range cerebello‐cortical communication but do not directly inform on the baseline physiological state of the connected cortical territories themselves.

In this framework, fALFF could provide a complementary perspective to support a diaschisis‐related physiological depression that would explain the functional repercussions of FA‐impacted cerebello‐cortical loops. Diaschisis, originally defined by von Monakow as a loss of function in intact brain regions secondary to a remote but connected lesion, has been refined by metabolic and neuroimaging studies demonstrating reductions in perfusion, glucose uptake, and spontaneous neural activity within structurally preserved but functionally disconnected territories (Carrera and Tononi 2014; Pantano et al. 1986). In FA, the severe degeneration of the dentate nuclei and disruption of the DTC tract provide a clear anatomical substrate for such downstream physiological depression. From this perspective, fALFF provides a complementary measure to functional connectivity by indexing the baseline amplitude of spontaneous BOLD fluctuations within individual regions, rather than their temporal synchrony with other areas. Physiologically, this allows the detection of regional reductions in baseline activity that may arise from loss of afferent cerebellar drive, even in situations where interregional functional connectivity is relatively preserved. In the context of FA, such fALFF reductions are therefore well suited to capture diaschisis‐related downstream effects of cerebellar efferent failure across motor, associative, and limbic circuits.

In this study, we use fALFF to test whether (i) FA exhibits fALFF reductions of intrinsic activity along the cerebello–thalamo–cortical system, (ii) regional fALFF levels relate to cerebellar motor and non‐motor symptoms severity, and finally whether (iii) fALFF levels display a specific pattern for the cerebellar cognitive‐affective syndrome in FA.

## 2 Methods

### 2.1 Participants

Adults with genetically confirmed FA were recruited at the HUB–Hôpital Erasme (Brussels, Belgium). Each patient was individually matched by age and sex with a healthy control who had no history of neurological disease, psychiatric disorder, or psychoactive substance use. All participants underwent a standardized clinical and imaging evaluation on the same day.

### 2.2 Clinical Assessments

The clinical battery included both motor and non‐motor evaluations. The age at symptom onset (ASO) corresponded to the age when the first motor difficulties were reported by the participant or a close relative. Disease duration (DD) was calculated as the time elapsed between ASO and the date of MRI acquisition. Cerebellar motor function was quantified using the Scale for the Assessment and Rating of Ataxia (SARA), an eight‐item clinical tool that measures gait, stance, sitting balance, speech, upper‐ and lower‐limb coordination, and alternating hand movements. Scores range from 0 (normal) to 40 (most severe ataxia) (Schmitz‐Hübsch et al. 2006). Cognitive and affective symptoms were examined with the Cerebellar Cognitive Affective Syndrome Scale (CCAS‐S). This screening battery comprises 10 brief tests exploring executive, visuospatial, linguistic, and affective domains (semantic and phonemic fluency, category switching, forward and backward digit span, cube copying, verbal recall, similarities, go/no‐go, and emotion recognition). Each subtest yields a raw score contributing to a total score between 82 (minimum passing values) and 120 (maximum performance). Although the total score provides a continuous index of cognitive functioning, the diagnosis of CCAS relies on the number of failed subtests: one failure suggests *possible* CCAS, two failures *probable* CCAS, and three or more *definite* CCAS. Because of the additive scoring scheme, individuals may obtain a relatively high total score (> 90) yet still meet criteria for definite CCAS if multiple subtests fall below threshold. For subgroup analyses, participants with FA were stratified according to the CCAS‐S. Participants were classified according to the validated CCAS‐S diagnostic criteria (Hoche et al. 2018). Individuals failing three or more CCAS‐S subtests were classified as having definite CCAS (FA‐Cog), whereas those failing fewer than three subtests (i.e., none, one, or two failed subtests) were classified as cognitively preserved (FA‐NoCog). Normality of continuous variables was assessed using the Shapiro–Wilk test. Normally distributed variables are presented as mean ± standard deviation (SD) and were compared using Student's *t*‐tests, whereas non‐normally distributed variables are presented as median (range) and were compared using Mann–Whitney *U*. Categorical variables were compared using the *χ* ^2^ test.

### 2.3 Ethical Approval and Consent

All participants provided written informed consent before enrollment. The study was approved by the Ethics Committee of the CUB Hôpital Erasme, Brussels (Reference: CCB B4062021000183). Procedures conformed to the Declaration of Helsinki and subsequent amendments.

### 2.4 Data Availability

The anonymized datasets generated and analyzed during this study will be made publicly accessible upon publication.

### 2.5 MRI Acquisition

All MRI scans were acquired on a hybrid 3T SIGNA PET–MRI system (GE Healthcare, Milwaukee, WI, USA) equipped with a 24‐channel head and neck coil. Resting‐state functional images were obtained using T2*‐weighted gradient‐echo echo‐planar imaging (GE‐EPI) covering the whole brain (TR = 3000 ms, TE = 34 ms, flip angle = 90°, field of view = 290 × 290 mm^2^, acquisition matrix = 96 × 96, 40 axial slices, voxel size = 2.9 × 2.9 × 3.0 mm^3^, 120 volumes). Four dummy scans preceded each run to allow steady‐state magnetization. Participants were instructed to remain still, stay awake, and maintain fixation on a central cross during the 6‐min resting‐state acquisition. High‐resolution 3D T1‐weighted anatomical images were also acquired for tissue segmentation, spatial normalization, and volumetric analyses using the vol2Brain pipeline (Manjón et al. 2022), which provides normalized estimates of intracranial, supratentorial, and cerebellar gray matter volumes from T1‐weighted MRI (brain volume general electric healthcare product with TR = 8.3 ms, TE = 3.1 ms, TI = 450 ms, flip angle = 12°, voxel size = 1 mm isotropic with field of view adjusted for whole‐brain coverage).

### 2.6 Preprocessing and fALFF Computation

Functional MRI data were processed using SPM12 (Wellcome Centre for Human Neuroimaging, London, UK) and the CONN functional connectivity toolbox (release 22.a; Whitfield‐Gabrieli and Nieto‐Castanon 2012). Preprocessing followed CONN's standard volume‐based pipeline and included slice‐timing correction, rigid‐body realignment and unwarping, co‐registration to the individual T1‐weighted image, segmentation, normalization to MNI space, and spatial smoothing using a 6‐mm full‐width‐at‐half‐maximum Gaussian kernel. Potential outlier volumes were identified using the artifact detection tools (ART) as scans with framewise displacement > 0.9 mm or global BOLD signal changes > 5 SDs and were modeled as nuisance regressors during denoising.  Denoising further included regression of motion parameters, white matter and cerebrospinal fluid (CompCor) components, and identified outlier volumes. Voxelwise fALFF was computed within CONN's voxel‐to‐voxel intrinsic activity framework (https://web.conn‐toolbox.org/fmri‐methods/voxel‐to‐voxel‐analyses). For each voxel, fALFF was defined as the ratio of BOLD signal power within the 0.01–0.10 Hz band to the total detectable frequency spectrum (Zou et al. 2008). This implementation is consistent with previous CONN‐based studies applying fALFF in clinical populations (Karavallil Achuthan et al. 2023).

Second‐level analyses were performed in CONN using the random field theory framework implemented in SPM for voxelwise inference. Group contrasts included (i) Controls versus FA, and (ii) FA‐Cog versus FA‐NoCog. Between‐group differences were tested using independent‐samples group contrasts within the second‐level general linear model, equivalent to two‐sample *t*‐tests while accounting for the included nuisance covariates. Individual voxelwise fALFF maps were entered as the dependent data, and the resulting voxelwise T‐maps represent the adjusted between‐group difference in fALFF at each voxel. Age, sex, mean head motion, and cerebellar gray matter volume were included as nuisance covariates. Group contrasts were selected to address two complementary questions: the controls versus FA comparison assessed disease‐related alterations in intrinsic activity, whereas the direct FA‐Cog versus FA‐NoCog comparison assessed cognitive subgroup‐related differences within FA. Separate FA‐Cog or FA‐NoCog versus controls contrasts were not used as primary analyses because they would not distinguish cognitive subgroup effects from the overall effect of disease. Within the FA group, voxelwise linear regression analyses assessed associations between fALFF and clinical measures (SARA and CCAS‐S). Individual voxelwise fALFF maps served as the dependent variable, with clinical scores entered as continuous predictors. These analyses were conducted across the whole brain rather than being restricted to regions showing significant group differences, allowing identification of brain regions in which intrinsic activity varied with clinical severity. To account for potential confounding effects, all models included age, sex, mean head motion, and cerebellar gray matter volume as nuisance covariates. Statistical maps were first thresholded using a voxel‐level cluster‐forming threshold of *p* < 0.001 (uncorrected). Clusters exceeding this threshold were then tested for significance using cluster‐level family‐wise error (FWE) correction based on random field theory, with significance defined as pFWE < 0.05. Peak‐level uncorrected *T* values are reported for descriptive purposes (Whitfield‐Gabrieli and Nieto‐Castanon 2012; Penny et al. 2007; Nieto‐Castanon 2020). Anatomical labeling of significant clusters was performed using the atlas tools embedded in CONN. Voxelwise results were corrected for spatial multiple comparisons using cluster‐level FWE correction as described above; however, no additional correction was applied for testing the two clinical measures (SARA and CCAS‐S).

## 3 Results

### 3.1 Participants

Twenty‐eight individuals with FA and 28 age‐ and sex‐matched healthy controls participated in the study. Twenty‐seven patients were homozygous for a GAA1 triplet expansion in the FXN gene, and one was compound heterozygous carrying a GAA1 expansion on one allele and a pathogenic point mutation on the other. Based on the CCAS‐S classification, 14 patients were assigned to the FA‐Cog subgroup and 14 to the FA‐NoCog subgroup. Structural volumetric analysis using the automated vol2Brain pipeline showed no significant differences in intracranial cavity volume (14.19 ± 1.27 vs. 13.65 ± 1.27 × 10^6^ mm^3^, *p* = 0.12) or supratentorial cortical gray matter volume (5.56 ± 0.52 vs. 5.49 ± 0.54 × 10^6^ mm^3^, *p* = 0.63) between controls and patients, whereas cerebellar gray matter volume was significantly reduced in patients (1.02 ± 0.20 vs. 1.14 ± 0.09 × 10^6^ mm^3^, *p* = 0.008); consequently, cerebellar gray matter volume was included as a second‐level nuisance covariate in all voxelwise analyses. Main demographic and clinical features are summarized in Table 1.

**TABLE 1:** Demographic, genetic, clinical, and cognitive characteristics of study participants.

Variable	FA (*n* = 28)	FA‐NoCog (*n* = 14)	FA‐Cog (*n* = 14)	p‐values	Controls (*n* = 28)
Age (years, mean ± SD)	32 ± 14	27 ± 10	37 ± 16	0.06	33 ± 7
F/M	16/12	8/6	8/6	—	16/12
GAA1 (mean ± SD)	672 ± 254	621 ± 311	722 ± 177	0.34	—
Age of symptom onset (ASO, mean ± SD)	17 ± 13	21 ± 14	13 ± 11	0.12	—
Disease duration (DD, mean ± SD)	14 ± 7	17 ± 8	12 ± 6	0.16	—
SARA (median [range])	12.5 (5–35)	17 (5–32)	28 (8–35)	0.15	—
CCAS‐S raw score (median [range])	90 (53–120)	101 (83–120)	83 (52–112)	**0.002**	115 (107–120)
CCAS‐S failed items (median [range])	2.5 (0–5)	2 (0–2)	4 (3–5)	—	0 (0–1)

*Note: p*‐values refer to comparisons between FA‐NoCog and FA‐Cog subgroups.

Abbreviations: ASO, age of symptom onset; CCAS‐S, Cerebellar Cognitive Affective Syndrome Scale; Controls, healthy control participants; DD, disease duration; FA, Friedreich ataxia; FA‐Cog, FA patients with cognitive impairment; FA‐NoCog, FA patients without cognitive impairment; GAA1, length of the shorter GAA trinucleotide repeat expansion; n, number of participants; SARA, Scale for the Assessment and Rating of Ataxia; SD, standard deviation.

#### 3.1.1 Whole‐Group Difference (Controls > FA)

No participant was excluded because of excessive head motion (Table 2; Figure 1). Mean framewise displacement was significantly higher in patients than in controls (0.173 ± 0.137 mm vs. 0.066 ± 0.047 mm, *p*  = 0.0003). To account for this difference, all voxelwise analyses were repeated including mean framewise displacement as a nuisance covariate in all analysis. Whole‐brain fALFF analysis identified three clusters surviving cluster‐level FWE correction, all reflecting significantly lower fALFF in patients with FA than in healthy controls (Table 1; Figure 1). No significant clusters were observed for the opposite contrast (FA > controls). The largest cluster was centered in the left frontal pole extending into the paracingulate and anterior cingulate cortices (peak MNI: −8, +46, +20; cluster size = 301 voxels; cluster‐level *p*FWE < 0.001). A second significant cluster was located in the left frontal pole (peak MNI: −30, +54, −4; cluster size = 179 voxels; cluster‐level *p*FWE = 0.012). A third cluster involved the medial superior frontal gyrus and paracingulate cortex (peak MNI: 0, +16, +42; cluster size = 137 voxels; cluster‐level *p*FWE = 0.04).

**TABLE 2:** Brain regions showing significantly lower resting‐state fALFF in patients with Friedreich ataxia than healthy controls.

Peak MNI coordinates (*x*, *y*, *z*)	Principal anatomical region	Cluster size (voxels)	Peak *T*	Cluster‐level pFWE
−8, 46, 20	Left frontal pole, paracingulate cortex, anterior cingulate cortex	301	5.18	< 0.001
−30, 54, −4	Left frontal pole	179	5.14	0.012
0, 16, 42	Medial superior frontal gyrus, paracingulate cortex	137	4.94	0.040

*Note*: Whole‐brain voxelwise comparison of resting‐state fALFF between healthy controls and patients with Friedreich ataxia (controls > FA). Statistical maps were thresholded using a voxel‐level cluster‐forming threshold of p < 0.001 (uncorrected), followed by cluster‐level family‐wise error (FWE) correction (pFWE < 0.05). Peak *T*‐values correspond to the voxel showing the maximal statistical effect within each significant cluster. Anatomical labels were assigned using the CONN atlas.

**FIGURE 1:**
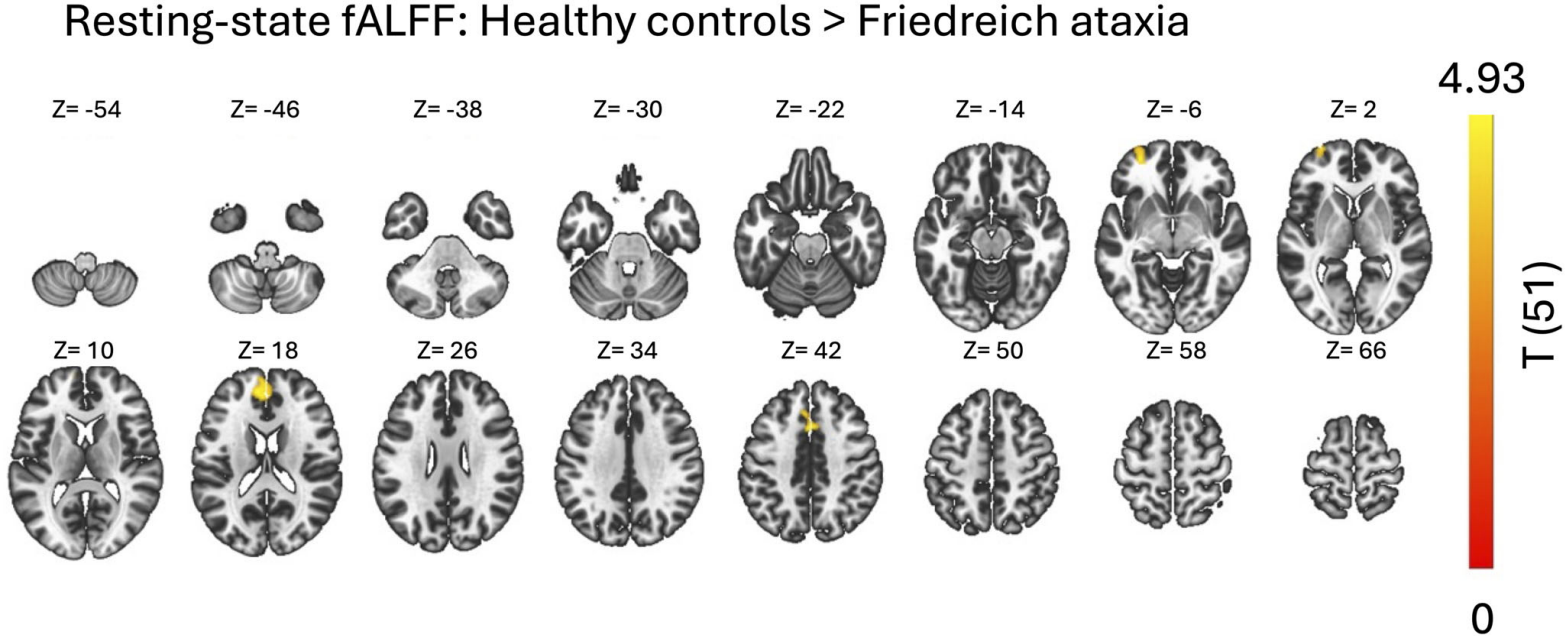
Brain regions showing significantly higher resting‐state fALFF in healthy controls than in patients with Friedreich ataxia (healthy controls > FA). Statistical maps are thresholded using a voxel‐level cluster‐forming threshold of *p* < 0.001 (uncorrected) and cluster‐level family‐wise error (FWE) correction at *p*FWE < 0.05. Images are displayed in MNI space using neurological convention. The color bar represents voxelwise T‐statistics.

#### 3.1.2 Contrast Between FA‐Cog and FA‐NoCog

Direct whole‐brain comparison between FA‐Cog and FA‐NoCog patients revealed two significant clusters surviving cluster‐level FWE correction (Tables 3 and 4; Figure 2). Compared with FA‐Cog patients, FA‐NoCog patients exhibited higher fALFF values within a large left fronto‐opercular cluster (peak MNI: −62, 10, 10; cluster size = 757 voxels; cluster‐level *p*FWE < 0.001), encompassing the middle frontal gyrus, inferior frontal gyrus, and precentral gyrus. A second significant cluster was identified in the right fronto‐insular region (peak MNI: 30, 4, 4; cluster size = 739 voxels; cluster‐level *p*FWE < 0.001), extending across the insular cortex, inferior frontal gyrus, frontal operculum, and putamen. No significant clusters were observed for the opposite contrast (FA‐Cog > FA‐NoCog). All reported findings survived cluster‐level FWE correction for multiple comparisons.

**TABLE 3:** Brain regions showing significantly lower resting‐state fALFF in patients with definite cerebellar cognitive affective syndrome than in cognitively preserved patients.

Peak MNI coordinates (*x*, *y*, *z*)	Principal anatomical region	Cluster size (voxels)	Peak *T*	Cluster‐level pFWE
−62, 10, 10	Left middle frontal gyrus extending into the inferior frontal gyrus and precentral gyrus	757	5.36	< 0.001
30, 4, 4	Right insular cortex extending into the inferior frontal gyrus, frontal operculum and putamen	739	5.73	< 0.001

*Note*: Whole‐brain voxelwise comparison of resting‐state fALFF between patients with definite cerebellar cognitive affective syndrome (FA‐Cog) and cognitively preserved patients (FA‐NoCog), showing regions with significantly lower fALFF in the FA‐Cog group. Statistical maps were thresholded using a voxel‐level cluster‐forming threshold of p < 0.001 (uncorrected), followed by cluster‐level family‐wise error (FWE) correction (pFWE < 0.05). Peak *T*‐values correspond to the voxel showing the maximal statistical effect within each significant cluster. Anatomical labels were assigned using the CONN atlas.

**TABLE 4:** Brain regions showing significant negative associations between resting‐state fALFF and SARA score in patients with Friedreich ataxia.

Peak MNI coordinates (*x*, *y*, *z*)	Principal anatomical region	Cluster size (voxels)	Peak *T*	Cluster‐level pFWE
26, −94, 22	Right occipital pole extending to bilateral lateral occipital cortex, intracalcarine cortex and cuneus	1673	−5.89	< 0.001
32, −46, 68	Right sensorimotor cortex	271	−5.67	0.001
26, −76, −42	Right cerebellar Crus II	188	−5.45	0.009
−4, −26, 28	Posterior cingulate cortex	130	−5.57	0.048

*Note*: Whole‐brain voxelwise regression analysis showing brain regions in which resting‐state fALFF was negatively associated with SARA score within the Friedreich ataxia group. Statistical maps were thresholded using a voxel‐level cluster‐forming threshold of p < 0.001 (uncorrected), followed by cluster‐level family‐wise error (FWE) correction (pFWE < 0.05). Peak *T*‐values correspond to the voxel showing the maximal statistical effect within each significant cluster. Anatomical labels were assigned using the CONN atlas.

**FIGURE 2:**
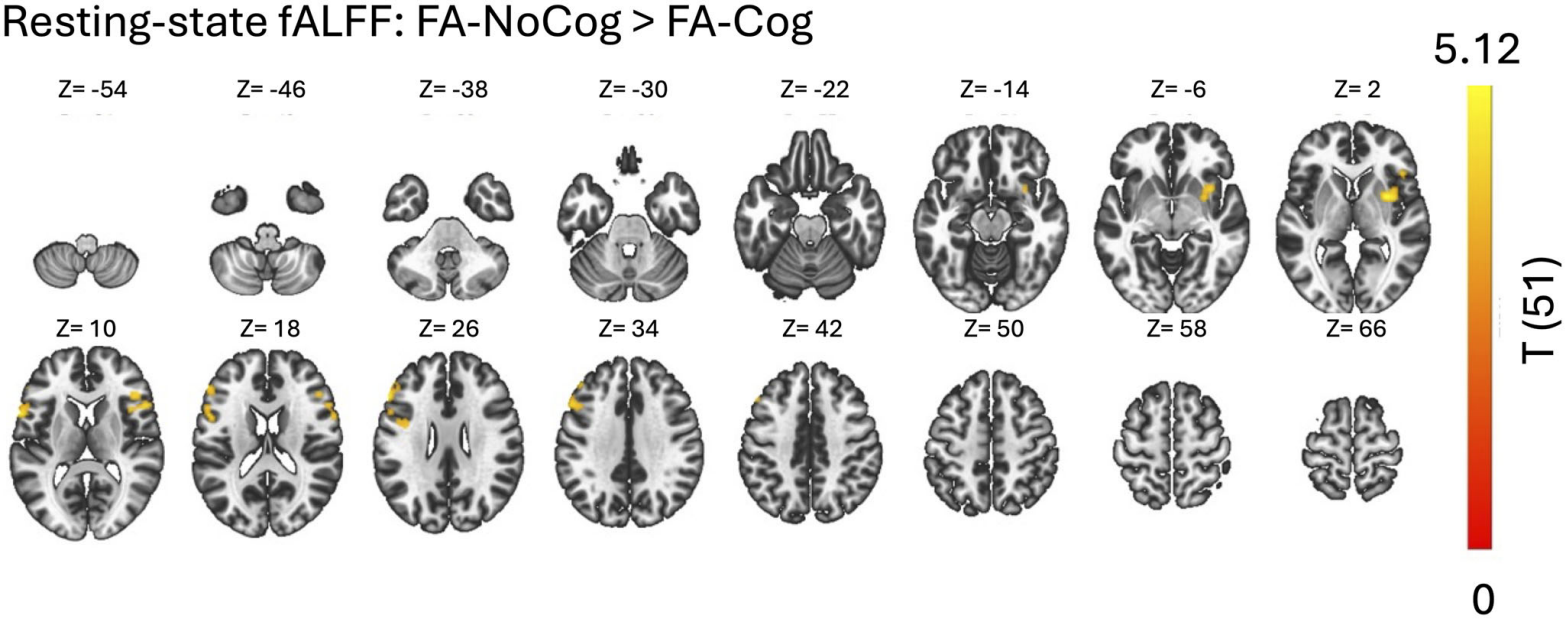
Whole‐brain comparison of resting‐state fALFF between cognitively preserved patients with Friedreich ataxia (FA‐NoCog) and patients with definite cerebellar cognitive affective syndrome (FA‐Cog). Brain regions showing significantly lower resting‐state fALFF in FA‐Cog than in FA‐NoCog (FA‐NoCog > FA‐Cog). Statistical maps are thresholded using a voxel‐level cluster‐forming threshold of *p* < 0.001 (uncorrected) and cluster‐level family‐wise error (FWE) correction at *p*FWE < 0.05. Images are displayed in MNI space using neurological convention. The color bar represents voxelwise T‐statistics.

#### 3.1.3 Clinical Correlations: SARA and CCAS‐S (Within FA)

Within the FA group, higher SARA scores were associated with significantly lower fALFF in four clusters surviving cluster‐level FWE correction (Tables 3, 4, 5; Figure 3). The largest cluster was centered in the right occipital pole (peak MNI: +26, −94, +22; cluster size = 1673 voxels; cluster‐level *p*FWE < 0.001), extending into the bilateral occipital pole, lateral occipital cortex, intracalcarine cortex and cuneus. Additional significant clusters were identified in the right sensorimotor cortex (peak MNI: +32, −46, +68; cluster size = 271 voxels; cluster‐level *p*FWE = 0.001), the right cerebellar Crus II (peak MNI: +26, −76, −42; cluster size = 188 voxels; cluster‐level *p*FWE = 0.009), and the posterior cingulate cortex (peak MNI: −4, −26, +28; cluster size = 130 voxels; cluster‐level *p* FWE = 0.048).

**TABLE 5:** Brain regions showing significant positive associations between resting‐state fALFF and CCAS‐S raw score in patients with Friedreich ataxia.

Peak MNI coordinates (*x*, *y*, *z*)	Principal anatomical region	Cluster size (voxels)	Peak T	Cluster‐level pFWE
4, 32, 58	Superior frontal gyrus, medial prefrontal cortex	447	5.43	< 0.001
32, −56, −22	Right cerebellar lobule VI and the temporo‐occipital fusiform cortex	439	5.72	< 0.001
−26, 44, 20	Left frontal pole	371	5.48	< 0.001
−14, −66, −48	Left cerebellar lobule VIII/Crus II	344	5.50	< 0.001
−44, −40, −14	Left inferior temporal gyrus and temporo‐occipital fusiform cortex	162	5.56	0.017

*Note*: Whole‐brain voxelwise regression analysis showing brain regions in which resting‐state fALFF was positively associated with CCAS‐S raw score within the Friedreich ataxia group. Statistical maps were thresholded using a voxel‐level cluster‐forming threshold of p < 0.001 (uncorrected), followed by cluster‐level family‐wise error (FWE) correction (pFWE < 0.05). Peak *T*‐values correspond to the voxel showing the maximal statistical effect within each significant cluster. Anatomical labels were assigned using the CONN atlas.

**FIGURE 3:**
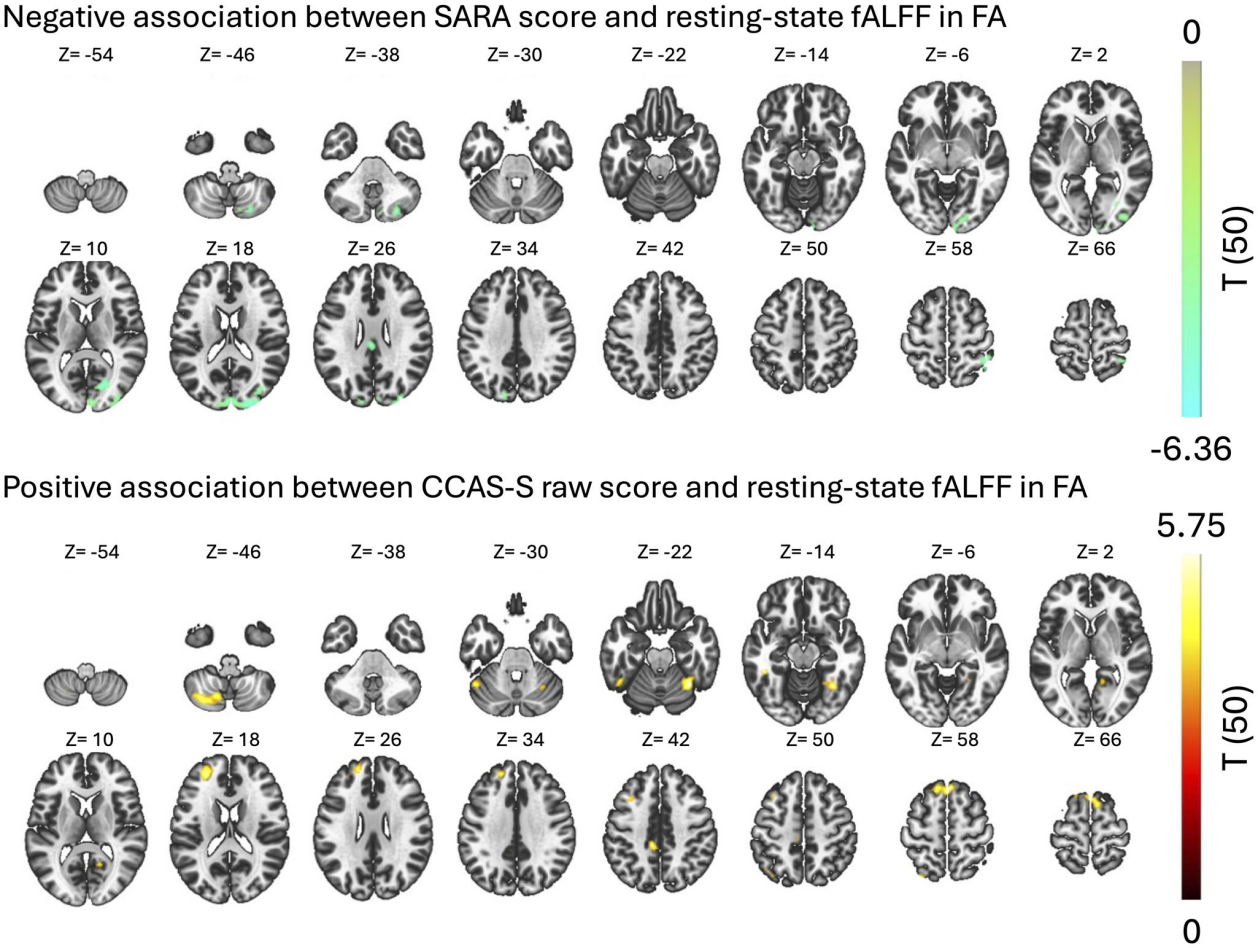
Whole‐brain voxelwise associations between resting‐state fALFF and clinical measures in patients with Friedreich ataxia. Top: Brain regions showing significant negative associations between SARA score and resting‐state fALFF. Bottom: Brain regions showing significant positive associations between CCAS‐S raw score and resting‐state fALFF. Statistical maps are thresholded using a voxel‐level cluster‐forming threshold of *p* < 0.001 (uncorrected) and cluster‐level family‐wise error (FWE) correction at *p*FWE < 0.05. Images are displayed in MNI space using neurological convention. Color bars represent voxelwise *T*‐statistics for the corresponding regression contrasts.

Higher CCAS‐S raw scores were associated with significantly higher fALFF in five clusters surviving cluster‐level FWE correction (Table 4). The largest cluster was centered in the superior frontal gyrus/medial prefrontal cortex (peak MNI: +4, +32, +58; cluster size = 447 voxels; cluster‐level *p*FWE< 0.001). Additional significant clusters were identified in the right cerebellar hemisphere involving lobule VI and the temporo‐occipital fusiform cortex (peak MNI: +32, −56, −22; cluster size = 439 voxels; cluster‐level *p*FWE< 0.001), the left frontal pole (peak MNI: −26, +44, +20; cluster size = 371 voxels; cluster‐level *p* FWE< 0.001), the left cerebellar hemisphere involving lobule VIII and Crus II (peak MNI: −14, −66, −48; cluster size = 344 voxels; cluster‐level *p*FWE< 0.001), and the left inferior temporal/temporo‐occipital fusiform cortex (peak MNI: −44, −40, −14; cluster size = 162 voxels; cluster‐level *p*FWE = 0.017). The reported *p*‐values for the associations with SARA and CCAS‐S were not additionally corrected for testing the two clinical measures

## 4 Discussion

The main findings of this study are threefold. First, after controlling for age, sex, head motion, and cerebellar gray matter volume, patients with FA exhibited reduced resting‐state fALFF predominantly within medial and lateral prefrontal regions, suggesting that functional alterations extend beyond structural cerebellar degeneration. Second, fALFF was significantly associated with clinical severity, with higher SARA scores correlating with reduced intrinsic activity in occipital cortex, sensorymotor cortex, and cerebellar regions, while higher CCAS‐S scores correlated with increased fALFF within bilateral prefrontal and cerebellar cognitive networks. Third, patients with clinically manifest CCAS exhibited additional reductions in fronto‐opercular and insular regions compared with cognitively preserved patients. Taken together, these findings support the concept that cerebellar degeneration in FA produces progressive dysfunction of structurally connected cerebral networks. In this framework, resting‐state fALFF may represent a functional marker of downstream cerebello‐cortical dysfunction compatible with cerebello‐cortical diaschisis, linking cerebellar pathology with downstream alterations in distributed motor and cognitive networks.

Although the cohort size is necessarily modest given the rarity of FA, the demographic and clinical characteristics of our participants, including age distribution, genetic profile, ASO, DD, and range of motor severity, closely mirror those reported in large multicenter natural history studies, such as the European Friedreich's Ataxia Consortium for Translational Studies (Reetz et al. 2021). In addition, cognitive performance assessed with the CCAS‐S in our cohort falls within the range previously described in independent FA populations, including large Australian and European cohorts, indicating comparable representation of non‐motor involvement (Destrebecq et al. 2023; Corben et al. 2024). Taken together, these facts suggest that the present sample captures the typical features of FA, supporting the generalizability of our results. Although the absolute CCAS‐S raw scores in our cohort were not markedly reduced, these findings remain clinically relevant. In the PROFA cohort, that included 101 patients with FA and reported comparable mean CCAS‐S raw score with also half of patients fulfilling criteria for definite CCAS, CCAS‐S raw score was independently associated with functional disability (Petit et al. 2026). Patient‐reported outcome studies in FA indicate that cognitive symptoms have a meaningful impact on daily life, being reported by approximately 40% of FA individuals (Seabury et al. 2023). These facts support CCAS as an intrinsic manifestation of FA rather than a secondary consequence of motor impairment. CCAS severity appears to lie on a continuum across cerebellar disorders, with FA generally exhibiting milder impairment than disorders such as SCA2 or MSA‐C, while still reflecting clinically relevant disruption of cerebello‐cortical networks (Petit et al. 2025; Destrebecq and Naeije 2023). Consistent with this interpretation, our neuroimaging findings demonstrate associations between resting‐state functional alterations within these networks and CCAS‐S performance, suggesting that even modest reductions in CCAS‐S scores reflect measurable dysfunction of cerebellar cognitive circuits rather than incidental findings.

These findings extend prior resting‐state connectivity studies in FA by demonstrating that cerebello‐cortical dysfunction is not limited to altered temporal synchrony between regions but is also characterized by a reduction in baseline intrinsic neural activity. Whereas previous rs‐fMRI investigations primarily emphasized disrupted coupling within motor and associative networks (Kerestes et al. 2023; Destrebecq et al. 2025), the present results indicate that these connectivity alterations are accompanied by decreased levels of fALFF, consistent with a diaschisis‐like process. The association between fALFF reductions in the sensorymotor cortex and ataxic motor severity further supports the interpretation of fALFF as a marker of physiological depression downstream of cerebellar efferent failure; notably, this pattern closely parallels findings from fluorodeoxyglucose positron emission tomography (FDG‐PET) studies in FA, which have shown that more severely affected patients exhibit lower cerebral glucose metabolism in cortical regions, consistent with progressive downstream functional deafferentation (Gilman et al. 1990). The association between reduced occipital fALFF and motor severity should be interpreted cautiously. One possible explanation is that these findings reflect downstream functional consequences of visual pathway involvement, which is increasingly recognized in FA. Optical coherence tomography (OCT) studies have consistently demonstrated retinal nerve fiber layer thinning and structural abnormalities along the anterior visual pathway, with several measures correlating with neurological severity. Similarly, recent work in spinocerebellar ataxia type 3 has shown that combined PERG/PVEP and OCT abnormalities are associated with disease severity, supporting the clinical relevance of retinal and visual electrophysiological biomarkers in hereditary ataxias (Liu et al. 2026). However, because visual acuity, OCT, PERG/PVEP, optic nerve imaging, and retinal measures were not acquired in the present study, this interpretation remains speculative and should be considered hypothesis‐generating rather than evidence of a direct relationship between retinal degeneration and occipital fALFF alterations. Visual system involvement provides a plausible explanation for the inverse relationship observed between fALFF and clinical severity in posterior occipital regions. In FA, retinal pathology is well established: OCT studies consistently demonstrate thinning of the retinal nerve fiber layer, which correlates with neurological severity, including SARA score, indicating that retinal and optic nerve degeneration parallels overall disease progression (Rodden et al. 2023; Thomas‐Black et al. 2023). Beyond FA, converging evidences from ocular and retinal disorders such as glaucoma, optic neuropathies, and retinal degeneration show that reduced afferent visual input is associated with decreased intrinsic activity in primary and associative visual cortices, as reflected by lower fALFF values on rs‐fMRI (Chai et al. 2025; Shi et al. 2025; Lai et al. 2022). Within this framework, the inverse correlation between occipital fALFF and motor ataxia severity likely reflects downstream physiological depression of visual cortical areas secondary to progressive retinal and optic pathway involvement, rather than a primary cortical process in FA.

Taken together, our findings also support a progressive model of cognitive involvement in FA in which cerebellar degeneration and impaired cerebello‐cortical communication contribute to downstream cortical dysfunction. The significant association between CCAS‐S raw scores and fALFF indicates that cognitive impairment varies continuously with intrinsic activity in prefrontal and cerebellar regions, while the direct FA‐Cog versus FA‐NoCog contrast shows that patients with clinically manifest CCAS exhibit additional reductions within frontal–opercular, insular, and striatal networks. This interpretation is consistent with prior evidence that reduced cerebello‐cerebral functional connectivity in FA correlates with disease severity and white matter damage in cerebellar efferent pathways (Destrebecq et al. 2023, 2025; Dadsena et al. 2026; Jing et al. 2026), and with previous finding that Crus I–prefrontal connectivity is associated with CCAS in this population. In line with the recent PROFA cohort showing that CCAS‐S performance correlates with clinical severity (Petit et al. 2026), these converging data suggest that cognitive decline in FA is not an isolated cognitive comorbidity, but part of a progressive cerebello‐cortical disconnection process. Within this framework, reduced fALFF may reflect a diaschisis‐like downstream cortical consequence of cerebellar degeneration, linking structural damage, disrupted cerebello‐cortical connectivity, and the emergence of cognitive symptoms.

Several limitations should be acknowledged. First, the cross‐sectional design precludes direct inferences about temporal progression or causality. Although the observed associations between fALFF reductions and clinical severity are consistent with a diaschisis‐related mechanism, longitudinal studies will be required to determine whether intrinsic activity changes precede, parallel, or follow clinical deterioration. Second, the present study did not include direct structural or metabolic measures, such as dentate nucleus volumetry, DTC tract microstructure, FDG‐PET, or retinal OCT, within the same individuals. Accordingly, interpretations regarding cerebellar efferent failure and visual pathway involvement rely on strong converging evidence from the existing FA literature rather than within‐subject multimodal correlations. Third, cognitive impairment was assessed using the CCAS‐S, a validated screening tool that captures the presence of CCAS but may be less sensitive to graded variations in cognitive performance. In addition, years of education were not systematically collected and could therefore not be included as a matching variable or covariate. As educational attainment may influence CCAS‐S performance, this may have contributed to between‐group differences and warrants cautious interpretation of the cognitive findings. Finally, because fALFF is derived from the BOLD signal, it reflects a composite measure influenced by neuronal activity as well as vascular and metabolic components of neurovascular coupling. Nonetheless, convergent PET–fMRI evidence (Aiello et al. 2015) and parallel findings in FA (Junck et al. 1994) and other neurodegenerative (Marchitelli et al. 2018) conditions support its relevance as an indirect marker of downstream neuronal physiological alterations arising from network disconnection. Still, large‐scale collaborative initiatives such as the ENIGMA‐Ataxia consortium (Harding et al. 2021) for fMRI data will be instrumental in addressing these limitations by enabling longitudinal analyses and substantially larger, harmonized samples, thereby improving statistical power, generalizability, and the integration of multimodal imaging biomarkers (e.g., structural, diffusion, functional) in FA.

In conclusion, the present study demonstrates that FA is associated with widespread reductions in intrinsic low‐frequency BOLD activity across cerebello‐cortical systems, extending beyond disrupted functional connectivity to encompass a depression of baseline neural activity consistent with a diaschisis‐like mechanism. By linking fALFF alterations to motor severity, cognitive phenotype, and involvement of sensorimotor, prefrontal, and visual cortices, our findings provide a unified physiological framework through which cerebellar efferent degeneration translates into distributed cortical dysfunction. These results position fALFF as a complementary and mechanistically informative marker bridging structural degeneration, network disconnection, and clinical expression in FA. Future longitudinal and multimodal studies, particularly within large collaborative frameworks, will be essential to establish the temporal dynamics of these changes and to evaluate the potential of fALFF as an outcome measure for disease monitoring and therapeutic interventions targeting cerebello‐cortical circuits.

## Author Contributions

**Naeije Gilles**: conceptualization, investigation, writing – original draft, methodology, writing – review and editing, formal analysis, data curation, supervision, validation, visualization, funding acquisition. **Trotta Nicola**: methodology, validation, writing – review and editing, data curation. **Rovai Antonin**: investigation, methodology, validation, visualization, writing – review and editing, data curation.

## Author Contributions

N.G. conducted the experiments, analyzed the data designed and conceptualized study, wrote and drafted the manuscript for intellectual content. R.A. analyzed the data and drafted the manuscript for intellectual content. T.N. conducted the experiments and drafted the manuscript for intellectual content.

## Ethics Statement

This study was conducted at the CUB Erasme Hospital (Brussels, Belgium) following approval by the institutional Ethics Committee (Reference: CCB B4062021000183). It was performed in accordance with the ethical standards laid down in the 1964 Declaration of Helsinki and its later amendments or comparable ethical standards. All participants provided written informed consent prior to inclusion.

## Conflicts of Interest

The authors declare no conflicts of interest.

## Data Availability

Anonymized data not published within this article will be made available by request from any qualified investigator and after acceptance of institutional (Université Libre de Bruxelles and Hôpital Universitaire de Bruxelles) authorities.
